# Barriers to access to treatment for mothers with postpartum depression in primary health care centers: a predictive model^1^

**DOI:** 10.1590/1518-8345.0982.2675

**Published:** 2016-03-28

**Authors:** Pablo Martínez, Paul A. Vöhringer, Graciela Rojas

**Affiliations:** 2 Doctoral student, Escuela de Psicología, Universidad de Santiago de Chile, Santiago, Chile. Assistant Researcher, Departamento de Psiquiatría y Salud Mental, Hospital Clínico, Facultad de Medicina, Universidad de Chile, Santiago, Chile; 3 PhD, Associate Professor, Departamento de Psiquiatría y Salud Mental, Hospital Clínico, Facultad de Medicina, Universidad de Chile, Santiago, Chile; 4 PhD, Full Professor, Departamento de Psiquiatría y Salud Mental, Hospital Clínico, Facultad de Medicina, Universidad de Chile, Santiago, Chile

**Keywords:** Depression, Postpartum, Health Services Accessibility, Primary Health Care

## Abstract

**Objective:**

to develop a predictive model to evaluate the factors that modify the access to treatment for Postpartum Depression (PPD).

**Methods:**

prospective study with mothers who participated in the monitoring of child health in primary care centers. For the initial assessment and during 3 months, it was considered: sociodemographic data, gyneco-obstetric data, data on the services provided, depressive symptoms according to the Edinburgh Postpartum Depression Scale (EPDS) and quality of life according to the Short Form-36 Health Status Questionnaire (SF-36). The diagnosis of depression was made based on MINI. Mothers diagnosed with PPD in the initial evaluation, were followed-up.

**Results:**

a statistical model was constructed to determine the factors that prevented access to treatment, which consisted of: item 2 of EPDS (OR 0.43, 95%CI: 0.20-0.93) and item 5 (OR 0.48, 95%CI: 0.21-1.09), and previous history of depression treatment (OR 0.26, 95%CI: 0.61-1.06). Area under the ROC curve for the model=0.79; p-value for the Hosmer-Lemershow=0.73.

**Conclusion:**

it was elaborated a simple, well standardized and accurate profile, which advises that nurses should pay attention to those mothers diagnosed with PPD, presenting low/no anhedonia (item 2 of EPDS), scarce/no panic/fear (item 5 of EPDS), and no history of depression, as it is likely that these women do not initiate treatment.

## Introduction

Postpartum depression (PPD) is a public health problem worldwide^(^ 1 ^)^. It is the most common psychiatric condition postpartum^(^ 2 ^)^ and there is extensive material on the degree of disability that it is likely to cause to the mother^(^ 3 ^)^, its association with the delay in child development and behavior disorders in adult life of the descendants^(^ 4 ^)^.

In Chile, studies using standardized diagnostic criteria reported a prevalence of PPD of about 20% in the primary health care (PHC) of public health system^(^ 5 ^)^. In contrast, a study using the Edinburgh Postpartum Depression Scale (EPDS), validated in Chile^(^ 6 ^)^, indicated that 41.3% of mothers who are assisted in clinics are affected by severe depressive symptoms between 2 and 3 months postpartum^(^ 7 ^)^, that is, at risk of PPD.

Although a significant proportion of mothers who use the APS are at high risk and the importance of maternal and child health leads to a greater number of visits to health centers in this period, depressive disorders are not usually detected and treated^(^ 8 ^)^, despite the availability of effective treatments^(^ 9 ^)^.

Based on that, the Ministry of Health^(^ 10 ^)^ promoted a early detection of PPD, recommending the adoption of the universal screening in the PHC, so that the EPDS is applied by nursing professionals in the follow-up of children and women at postpartum period. However, treatment rates remain low.

In this regard, the national literature has evidenced the presence of barriers to access to health services for depressed mothers and the need for trainnig of human resources in the PHC in order to ensure a greater commitment to the ministerial guidelines and tighter monitoring of women at risk^(^ 11 ^)^.

It is considered that the construction of a predictive model to identify the factors that modify the access to treatment may be useful in reducing the failures in the treatment of PPD, by focusing on the use of human resources available in the public health system, and specifically, strengthening the role of nurses in detecting PPD during routine examinations.

There are no studies in the local context that have investigated that aspect at present.

The aim of this study was to develop a predictive model to evaluate the factors that modify the access to treatment for PPD in PHC.

## Method

This is a prospective cohort study. The sampling consisted of all health units of PHC located in the Metropolitan Region (MR), Chile (n=120). It was selected the health unit of PHC that registered the highest number of health attendances of children in the past 2 months, in each of the six Health Services of the MR, according to administrative data of the Ministry of Health, in the period from January to September, 2012. In this way, the sample consisted of six municipal health units of PHC of the MR, Chile. This due to the fact that the administrative data from the Ministry of Health are not broken down by month.

During the months of January and February 2013, it was consecutively recruited those mothers participating in the child health monitoring, from two to six months postpartum, at the selected health units. After routine examination, the study researchers included those mothers that have signed an informed consent, over 18 years old, without intellectual disability and could be contacted by telephone. All the women agreed to participate voluntarily.

A week later, a structured interview was carried out by phone (initial diagnosis), which assessed: sociodemographic antecedents, gynecological-obstetric and perinatal data, depressive symptoms, according to the Edinburgh Postpartum Depression Scale (EPDS)^(^ 7 ^)^, confirmation of current diagnosis of Major Depressive Postpartum Episode (PPD), according to the structured psychiatric interview MINI^(^ 12 ^)^ and quality of life, according to the SF-36 Health Status Questionnaire^(^ 13 ^)^.

The final sample used for collection and analysis of data in this study included only women in which PPD has been confirmed, according to MINI, in the initial diagnosis.

## Definition of dependent variable

After three months, the medical records of users with PPD (follow-up evaluation) were reviewed, considering as no access to treatment: if no provision of mental health consultation was recorded in the health unit after the initial diagnosis (dichotomized variable).

## Definition of independent variables

To determine the predictors of no access to treatment in women with PPD in PHC, a review of the available literature was performed^(^ 14 ^-^ 21 ^)^. Accordingly, the following variables were selected as potential predictors: age, marital status, education, current occupational status, who lives in the household, number of children, planning of the last pregnancy, help in caring for the baby, history of previous treatments of depression, depressive symptoms (total score of EPDS and score in each item of the instrument) and quality of life (according to the dimensions of the SF-36).

All variables that were significant with p<0.1 in the univariate analysis, were included in the multivariate model using a backward selection technique (*backward*), to obtain the most parsimonious multivariate predictive model. The Hosmer-Lemeshow test was used to measure the effectiveness of the predictive model, that is, the matching between the predicted and observed probabilities. To evaluate the discrimination ability of the model, that is, the probability to identify a case of PPD from a couple of observations taken at random, it was used the area under the ROC curve (*Receiver Operating Characteristics*). Statistical analyzes were performed with Stata 12.0^(^ 22 ^)^. All estimates were presented collectively with confidence intervals at 95% (95%CI).

## Results

The initial sample consisted of 305 women. In the initial diagnosis, PPD was confirmed in 63 of them (20.7%), which formed the final sample for the analysis. In the follow-up evaluation, it was possible to access the medical records of all women in the final sample, therefore, there was no loss of data.

As shown in Table 1, participants with PPD had a mean age of 27.6 years (Standard Deviation [SD] of 6.5 years), most were single (58.7%, 95%CI: 46.2-71.2) and had completed high school (50.8%, 95%CI: 38.1-63.5). At the time of evaluation, 47.6% (95%CI: 34.9-60.3) lived at home with a partner, and more than half were devoted to domestic tasks (60.3%, 95%CI: 47.9-72.7). Almost half (46%; 95%CI: 33-59) of women admitted to having been treated for previous depressive episodes. Of the 63 women with PPD, 79.4% (95%CI: 69.1-89.6) had not started the treatment after three months.

**Table 1:** - Sociodemographic and clinical characteristics of the sample, grouped according to the type of access to treatment. Santiago, Metropolitan Region, Chile, 2012-2013*^†^

**Variable**	**Total sample analyzed(n = 63)**	**Access to treatment20.6% (n = 13)**	**No access to treatment79.4% (n = 50)**	**Difference between means or RR (95%CI) ^‡^**	
Age (years)	27.6 (6.5)	29.6 (6.9)	27.1 (6.4)	2.56 (-1.47, 6.58)	
Number of children	2.2 (1.1)	2.4 (1.1)	2.1 (1.1)	0.28 (-0.41, 0.98)	
Marital status	Single	37 (58.7)	9 (24.3)	28 (75.7)	0.89 (0.70, 1.14)
	Cohabitant	8 (12.7)	0 (0)	8 (100)	1.31 (1.13, 1.52)
	Married	11 (17.5)	3 (27.3)	8 (72.7)	0.90 (0.61, 1.32)
	Separated	7 (11.1)	1 (14.3)	6 (85.7)	1.09 (0.78, 1.52)
Education	Incomplete elementary school	4 (6.3)	1 (25)	3 (75)	0.94 (0.53, 1.68)
	Complete elementary school	5 (7.9)	2 (40)	3 (60)	0.74 (0.36, 1.53)
	Incomplete high school	10 (15.9)	2 (20)	8 (80)	1.01 (0.72, 1.42)
	Complete high school	32 (50.8)	4 (12.5)	28 (87.5)	1.23 (0.95, 1.60)
	Higher	12 (19)	4 (33.3)	8 (66.7)	0.81 (0.53, 1.23)
Current occupation	Housewife	38 (60.3)	9 (23.7)	29 (76.3)	0.91 (0.71, 1.16)
	Student	1 (1.6)	0 (0)	1 (100)	1.27 (1.11, 1.44)
	Employee	23 (36.5)	3 (13)	20 (87)	1.16 (0.91, 1.47)
	Unemployed	1 (1.6)	1 (100)	0 (0)	0
Lives with	Partner	30 (47.6)	3 (10)	27 (90)	1.29 (1.00, 1.67)
	Parents	19 (30.2)	6 (31.6)	13 (68.4)	0.81 (0.58, 1.13)
	Alone with children	8 (12.7)	2 (25)	6 (75)	0.94 (0.62, 1.43)
	Others	6 (9.5)	2 (33.3)	4 (66.7)	0.83 (0.46, 1.48)
Planned pregnancy	19 (30.2)	1 (5.3)	18 (94.7)	1.30 (1.06, 1.61)	
Receives help to care for the baby	39 (61.9)	32 (82.1)	7 (17.9)	0.91 (0.70, 1.20)	
Previous treatments of depression	29 (46)	9 (31)	20 (69)	0.78 (0.59, 1.03)	
EPDS (total score)^§^	16.4 (4.4)	18.3 (4.7)	15.9 (4.2)	2.39 (-0.27, 5.05)	
Physical component summary^||^	47.1 (10)	43.2 (8.7)	48.1 (10.2)	-4.83 (-10.99, 1.33)	
Mental Component Summary^||^	24.4 (10.6)	22.9 (5.5)	24.8 (11.6)	-1.88 (-8.5, 4.75)	

The following variables were included in the predictive multivariate logistic regression model, which achieved statistical significance (p<0.1) in univariate evaluation: planning of pregnancy, history of previous treatment of depression, total score of EPDS, item 2 of EPDS ("anhedonia during the last week"), item 5 of EPDS ("panic or fear during the last week"), physical functioning dimension of the SF-36 and general health dimension of the SF-36.

After applying the backward technique for selection of the variables, the final model included the following factors that hindered the access to treatment:

1. Previous history of treatments of depression.
2. Item 2 of EPDS, presence of anhedonia in the last week.
3. Item 5 of EPDS, presence of panic or fear in the last week.

It is observed that together, the variables correctly classified 82.5% of the total cases, characterized by having a high sensitivity (96%), specificity of 30.8%, high positive predictive value (PPV) of 84.2% and a good negative predictive value (NPV) of 66.7%.

In assessing the behavior of the predictive model in the sample, with a prevalence of no access to treatment 79.4%, it was achieved a PPV of 91.9%, demonstrating a high probability of women experiencing depression, this is, without access to treatment, as predicted by the set of variables. On the other hand, the VPL obtained was low (38.5%), suggesting that the negative result in the predictive model is limited to determine if mothers with PPD have access to treatment.

**Figure 1:**
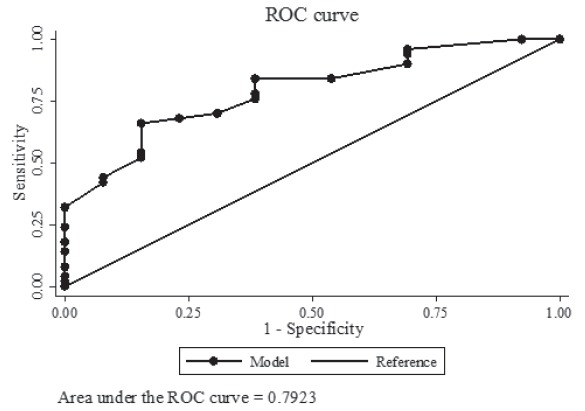
- Discriminatory ability of the adjusted multivariate logistic regression model

The power of the model is good, since the degree of discrepancy between the predicted and observed probabilities did not reach significant levels in the Hosmer-Lemeshow test (p=0.73), and the area under the ROC curve (auROC=0.79) suggests a good power of discrimination (Figure 1).

In the final model (Table 2), one-point increase in the second item of EPDS ("anhedonia") decreased by 57% (odds ratio [OR], 0.43; 95%CI: 0.20-0.93) the probability of *no access* to treatment. Similarly, one-point increase in item 5 of EPDS ("panic/fear") decreased in over half (OR 0.48, 95%CI: 0.21-1.09) the probability of *no access* to treatment. Finally, having as positive the antecedents of previous treatments of depression, decreased by 74% (OR 0.26, 95%CI: 0.61-1.06) the probability of *no access* to treatment, when compared with women who did not have previous treatments of depression.

**Table 2:** - Predictive multivariate logistic regression model. Santiago, Metropolitan Region, Chile, 2012-2013

**No access to treatment**	OR*	**95%CI^†^ for OR**	**P value**	
		**Lower**	**Higher**	
Item 2 EPDS^‡^: anhedonia	0.43	0.20	0.93	0.033
Item 5 EPDS: panic/fear	0.48	0.21	1.09	0.079
History of previous treatment of depression	0.26	0.61	1.06	0.061
Constant	135.19	8.66	2111.62	0.000

*Odds Ratio †Confidence Interval 95% ‡Edinburgh Postpartum Depression Scale

Although the latter two predictors were not statistically significant, they were "forced" into the model due to their contribution to a more parsimonious development of the predictive model and also based on the literature, which supported their inclusion^(^ 15 ^-^ 17 ^,^ 21 ^)^.

## Discussion

This is the first study in the national literature to develop a predictive model to evaluate the factors influencing the access to treatment for PPD in mothers who use PHC. Access to treatment of women with PPD is still very low, despite the existence of universal access and the availability of effective treatments.

According to this study, women who develop PPD and with no access to treatment are those presenting low levels of anhedonia and symptoms of anxiety (panic and fear), and who did not have prior history of treatment due to episodes of depression.

The model developed is simple (consisting of only three factors), shows good standardization and ability to discriminate. It is worth mentioning its high sensitivity (96%), indicating that the variables included are capable, as a whole, to predict properly, women who have no access to treatment.

It must be considered that its predictive value is quite sensitive to the prevalence of the event. Here, the high prevalence of the condition studied (no access to treatment) is reflected in the high PPV shown by the model, suggesting that if the set of variables predicts that the event will occur, it is likely that mothers do not have access to treatment. Hence, this kind of knowledge among nurses can be useful, by foreseeing the event and informing the health team.

The features mentioned above suggest that the model has potential applicability to solve failures in the treatment of PPD in PHC, as evidenced by recent studies^(^ 8 ^)^, therefore, it is relevant to public health and to the role played by nursing professionals at postpartum.

However, the practical significance of these findings must be viewed with caution. This study is a secondary analysis of databases on a research that was developed for other purposes, which imposes important limitations: it is likely that eventually significant predictors have not been included, since access to treatment of postpartum depression has been described as a complex phenomenon that involves not easily quantifiable variables such as domestic workload, the ideals of motherhood and the stigma associated with mental health problems^(^ 12 ^,^ 17 ^)^; In addition, the analyzes were performed based on a small sample (n=63), which could affect the power of the study.

However, the non-inclusion of variables consideres as difficult to measure ("complex") is related with the development of a pragmatic risk profile, relatively easy to use and which does not require an additional effort from the nursing profesional in PHC. This is not a matter of dismissing important topics for addressing the PPD (and maternal health, in general) such as domestic workload, ideals of motherhood and stigmas associated to mental health, however, the design of strategies aimed at that purpose requires aditional and intersectoral investigation.

In addition, it is worth mentioning that the variables included in the risk profile (score in the items 2 of EPDS -anhedonia- and 5 -panic and fear- and history of previous treatment of depression) found support in the literature, which reports that access to treatment for depression is associated with depressive symptom levels (or degree of disability) and history of treatment of the disease^(^ 14 ^-^ 16 ^,^ 20 ^)^.

It is legitimate to think that the predictive model developed could represent a valuable contribution to guide the decision-making of nursing profesionals in identifying profiles of mothers with PPD at high risk of not having access to treatment, based on the antecedents already available and/or easily accesible.

For example, self reporting is generally considered reliable as antecedent of previous treatment of depression, in cases in which this information is not registered in the medical records^(^ 23 ^)^. In the case of the scoring obtained by mothers in the items 2 and 5 of EPDS, it is worth mentioning that nursing professionals perform an universal screening using this instrument at postpartum monitoring of child health, at which time it is investigated the suspected of PPD, representing an opportunity to access treatment for the disease^(^ 10 ^)^.

Therefore, the use of this risk profile does not imply an additional or different workload of the one that has already been implemented in PHC, allowing the use of resources of the public health system and implementation of strategies to facilitate access to treatment in this population of mothers, at this critical moment.

In this regard, the literature emphasizes the need for training of PHC teams in managing the PPD, considering as important the establishment of referral protocols in cases in which the screening results are indicative of suspicion of the disease. This involves to properly inform the mothers about their possible depression, motivate them to adhere to treatment and prioritize the availability of hours for care^(^ 11 ^,^ 24 ^)^.

## Conclusion

In conclusion, it is considered that this study opens up a wide field for further research aiming at the establishment of a risk profile for the lack of access to treatment for women with PPD in PHC. This is a pragmatic predictive model that could guide the human resources available at PHC, to support the implementation of activities aimed at filling the gaps in the treatment of a disease, which has been recognized as a public health problem. In the same vein, it is suggested that nurses be attentive to those mothers with PPD that have low anhedonia, or lack thereof, without panic or fear and no history of depression, since these are the patients who are more likely to not start the treatment for the disease, according to the model. Further studies are needed to validate and evaluate the impact of using this risk profile in real clinical settings.
